# BMP-SMAD signalling output is highly regionalized in cardiovascular and lymphatic endothelial networks

**DOI:** 10.1186/s12861-016-0133-x

**Published:** 2016-10-10

**Authors:** Karen Beets, Michael W. Staring, Nathan Criem, Elke Maas, Niels Schellinx, Susana M. Chuva de Sousa Lopes, Lieve Umans, An Zwijsen

**Affiliations:** 1 VIB Center for the Biology of Disease, VIB, Leuven, Belgium; 2 Department of Human Genetics, KU Leuven, Leuven, Belgium; 3 Department Anatomy and Embryology, Leiden University Medical Centre, Leiden, The Netherlands; 4 Department of Development and Regeneration, KU Leuven, Leuven, Belgium

**Keywords:** BMP-SMAD signalling, Sprouting angiogenesis, Valve development, Lymphangiogenesis, Endocardium, Phenotype switching, Heterogeneity, Retina, Stochastic expression, Morphogen

## Abstract

**Background:**

Bone morphogenetic protein (BMP) signalling has emerged as a fundamental pathway in endothelial cell biology and deregulation of this pathway is implicated in several vascular disorders. BMP signalling output in endothelial cells is highly context- and dose-dependent. Phosphorylation of the BMP intracellular effectors, SMAD1/5/9, is routinely used to monitor BMP signalling activity. To better understand the in vivo context-dependency of BMP-SMAD signalling, we investigated differences in BMP-SMAD transcriptional activity in different vascular beds during mouse embryonic and postnatal stages. For this, we used the *BRE*::*gfp* BMP signalling reporter mouse in which the BMP response element (BRE) from the *ID1*-promotor, a SMAD1/5/9 target gene, drives the expression of *GFP*.

**Results:**

A mosaic pattern of GFP was present in various angiogenic sprouting plexuses and in endocardium of cardiac cushions and trabeculae in the heart. High calibre veins seemed to be more *BRE*::*gfp* transcriptionally active than arteries, and ubiquitous activity was present in embryonic lymphatic vasculature. Postnatal lymphatic vessels showed however only discrete micro-domains of transcriptional activity. Dynamic shifts in transcriptional activity were also observed in the endocardium of the developing heart, with a general decrease in activity over time. Surprisingly, proliferative endothelial cells were almost never GFP-positive. Patches of transcriptional activity seemed to correlate with vasculature undergoing hemodynamic alterations.

**Conclusion:**

The *BRE*::*gfp* mouse allows to investigate selective context-dependent aspects of BMP-SMAD signalling. Our data reveals the highly dynamic nature of BMP-SMAD mediated transcriptional regulation in time and space throughout the vascular tree, supporting that BMP-SMAD signalling can be a source of phenotypic diversity in some, but not all, healthy endothelium. This knowledge can provide insight in vascular bed or organ-specific diseases and phenotypic heterogeneity within an endothelial cell population.

**Electronic supplementary material:**

The online version of this article (doi:10.1186/s12861-016-0133-x) contains supplementary material, which is available to authorized users.

## Background

The formation of the cardiovascular and lymphatic network is crucial for development and physiology. The cardiovascular system fuels nearly every tissue with oxygen and nutrients and removes waste products, while the lymphatic system is important for the drainage of extravasated fluid, the uptake of fat and is a vital part of the immune system [1]. Blood vessel development by sprouting from pre-existing vessels is called sprouting angiogenesis. In hypoxic environments angiogenic sprouts with tip and stalk cells emerge. Sprouts anastomose to form new functional vessels that supply oxygen to the initially hypoxic environment [1]. From the cardinal vein some venous endothelial cells (ECs) differentiate into lymphatic ECs (LECs), that migrate to form lymphatic sacs which in turn sprout to form a lymphatic network similar to angiogenesis events [2]. Failure to establish a (lymphatic) vascular network leads to severe embryonic defects at mid-to late gestation, whereas misregulation after birth can lead to diseases such as cancer, chronic and inflammatory disorders and oedema [3–5].

ECs form the inner cellular lining of blood and lymphatic vessels and the heart, and differ in protein expression, morphology and function depending on the vascular bed. Exposure to external and internal cues as well as epigenetic programming results in EC macro-heterogeneity and micro-heterogeneity [6–8]. This means that the endothelium acquires site- and organ-specific structural and functional properties, which are extensively reviewed in Aird et al. [7–9].

BMP signalling has emerged as a fundamental pathway of EC identity by regulating cardiovascular and lymphatic development [10]. BMPs are members of the transforming growth factor beta (TGFβ) family with more than 20 BMP members identified. BMP ligands reported to function in ECs are BMP2/4/6/7/9/10 [11]. BMPs bind to heteromeric transmembrane receptor complexes that consist of type I (ALK1/2/3/6) and type II receptors (BMPR2, ACTR2A, ACTR2B) and often also a co-receptor (Endoglin, Betaglycan). Ligand binding and phosphorylation of the GS-domain of the type I receptor by the type II receptor leads to recruitment and phosphorylation of the intracellular effectors SMAD1, SMAD5 and SMAD9 (pSMAD1/5/9) [12]. SMAD9 is also known as SMAD8. Activated pSMADs form a complex with the common SMAD, SMAD4, and translocate to the nucleus where they stimulate transcription of specific BMP target genes such as the inhibitors of differentiation (*IDs*), *HEY1* and *SMAD6*/*7*; and repress e.g. *Apelin* [13]. BMPs can also regulate other (non-canonical) pathways that do not involve SMAD proteins [14, 15].

BMP signalling is highly tuned by extracellular and intracellular modulators, but also by signalling interplay with other signalling pathways. Furthermore, BMPs are known to trigger expression of different target genes in a dose-dependent manner [16, 17], a landmark of morphogens. In addition, hemodynamic changes can induce BMP signalling and activate SMAD proteins in ECs [18, 19]. Recently, excessive BMP6 has been implicated in cerebral cavernous malformation [20]. Moreover, other regionalized vascular disorders such as hereditary hemorrhagic telangiectasia (HHT) and pulmonary arterial hypertension (PAH) are mainly caused by mutations in the BMP receptors *ACVRL1* (encoding ALK1) or *ENG* (encoding Endoglin) and *BMPR2* respectively [21–24]. The question remains how mutations in components of the same BMP pathway can cause such organ-specific diseases. A better understanding of the heterogeneity in BMP signalling output in different vascular beds may provide this insight and perhaps even the opportunity for disease-specific therapy.

Phosphorylated SMAD1/5/9 are routinely used to monitor BMP transcriptional activity, however this may confound interpretation, because pSMADs also play a role in chromatin remodelling and miRNA biogenesis [15]. To investigate the transcriptional activity of BMP-SMAD signalling many BMP reporter mice have been generated [25–30]. In this study we examined the *BRE*::*gfp* reporter mouse in which BMP response elements (BRE), derived from the *ID1*-promotor, drive the expression of enhanced green fluorescent protein (eGFP) [25]. The substantial decrease in GFP levels observed in *Smad5*-deficient *BRE*::*gfp* embryos corroborate the BMP-SMAD sensitivity of this reporter [25]. A commonality between all BRE-based reporters is that BRE activity does not completely overlap with pSMAD1/5/9 signalling domains [26, 27, 29, 31] because the onset of reporter activity first requires *de novo* mRNA and protein synthesis and GFP maturation, and the half-life of the reporter protein may deviate from pSMAD1/5/9 [29, 32, 33]. Moreover, pSMAD1/5/9 can also bind with different affinities and regulate other DNA-sequences like e.g. MEME2 [34]; pSMAD1/5/9 also has non-transcriptional functions [15]. Additionally, the *BRE*::*gfp* reporter is heterozygous, and it is becoming apparent that gene expression in general occurs with bursts of monoallelic expression instead of constant biallelic expression [35, 36]. Nonetheless, the relevance of the *BRE*::*gfp* reporter mouse became apparent in our previous study. Discrete GFP localisation patterns in angiogenic endothelium of *BRE*::*gfp* embryos, with an otherwise widespread pSMAD1/5/9 localisation, singled out those cells that underwent ID-mediated BMP-SMAD and Notch co-signalling essential for robust stalk cell fate [37].

In this study we aimed to further document regional differences in BMP-SMAD dependent transcriptional activity in murine endothelium of blood vessels, lymphatic vessels and the heart at embryonic and postnatal stages. We defined regions with stereotypic mosaic and continuous *BRE*::*gfp* localisation patterns, yet also GFP-negative regions were found in areas where BMP-SMAD signalling has been reported, compatible with the morphogen functions of BMP ligands. Our data support that BMP-SMAD signalling can play a role in phenotype switching and endothelial cell heterogeneity.

## Methods

### Mice and tissue collection

*BRE*::*gfp* transgenic mice and endothelium-specific *Smad1*;*Smad5* knockout (*Tie2cre* ^+/*0*^;*Smad1* ^*fl*/*fl*^;*Smad5* ^*fl*/*fl*^) mice were used. Genotyping of transgenic mice was done as described [25, 37]. All embryos and postnatal organs were dissected in ice-cold diethylpyrocarbonate (DEPC)-treated phosphate buffered saline (PBS) and fixed overnight (ON) in 4 % paraformaldehyde (PFA) in PBS at 4 °C. Afterwards they were rinsed with PBS and saline and stored in 70 % ethanol until processing.

Fixed embryos of embryonic day (E) 9.5–12.5, E14.5 and E16.5 and P6 intestines were processed for paraffin sectioning. Skin tissue from E14.5 and E16.5 *BRE*::*gfp* embryos was dissected after fixation. Layers of muscle and tissue were carefully removed from the skin, leaving the superficial lymphatic network intact. From each embryo two skin biopsies were harvested. Retinas were collected from fixed eyes by removing the cornea and carefully lifting the retina from the remaining eyeball. Ears were collected from postnatal pups and separated into a ventral and dorsal side of which the latter was analysed. For each analysis a minimum of three animals was examined.

### Immunofluorescence

#### Whole mount procedure

Embryos, skin biopsies, retinas, mesentery and ear skins were rehydrated and blocked in 2 % bovine serum albumin (BSA) in Tris buffered saline (TBS) for 3 h at room temperature (RT). Tissues were incubated ON with primary antibodies in 2 % BSA in TBS at 4 °C, except for the embryos which were kept at RT. This was followed by blocking for 3 h in 2 % BSA in TBS and incubation with the secondary antibody ON (Alexa antibodies, Jackson Immunology). The list of primary antibodies and the used dilutions are provided in supplementary material (Additional file 1: Table S1).

After whole mount immunostaining of E9.5 (22 ± 2 somites) and E10 (30 ± 2 somites) embryos the forebrain and the abdomen caudally from the forelimb bud were transversally removed. All ventral tissues including the heart were removed and the neural tube was then cut open at the ventral side. The hindbrain was mounted on a glass slide with the ventral side facing up.

The mesentery was excised from the intestines after whole mount immunostaining, and the retina was cut into a four-leaf clover before mounting on a glass slide.

#### Paraffin sections

Transversal and sagittal sections (6–8 μm) of paraffin embedded tissues were processed for immunodetection using an automated platform (Ventana Discovery Ultra, Roche). Immunofluorescent triple detection of pSMAD1/5/9, GFP and MF20 was done manually. The list of primary antibodies, as well as the conditions used, are provided in supplementary material. Antigen retrieval was done by submerging the slides in Tris-EDTA buffer (10 mM Tris Base, 1 mM EDTA, 0.01 % Tween20, pH9.0) for 30 min at 96 °C. For pSMAD1/5/9, endogenous peroxidases were inactivated in 3%H_2_O_2_ in Methanol for 30 min and the antibody signal was amplified using the Perkin Elmer TSA Biotin system kit (NEL700A001KT).

### In situ hybridisation

Embryos were dissected in DEPC-treated PBS and fixed ON in 4 % PFA in PBS at 4 °C. Afterwards they were washed three times 30 min in DEPC-treated PBS, immersed in 15 % sucrose and snap frozen in Optimal Cutting Temperature (OCT) compound (Richard-Allan Scientific #6502) with liquid nitrogen. The *GFP* fluorescent in situ hybridisation (ISH) probe was custom designed with the probe designer tool from Stellaris (LGC biosearch technologies). The coding sequence of the *pEGFP*-*N2* plasmid (accession number U57608.1) was used for probe design.

Fluorescent ISH (FISH) was performed according to the manufacturer’s protocol (Stellaris) with the addition of a permeabilisation step with 1 % TritonX100 (Sigma T8787) in PBS. Images were acquired using a Nikon A1R Eclipse Ti confocal microscope.

## Results

### Co-localisation of *BRE*::*gfp* transcriptional activity and GFP in endothelium

**Fig. 1:**
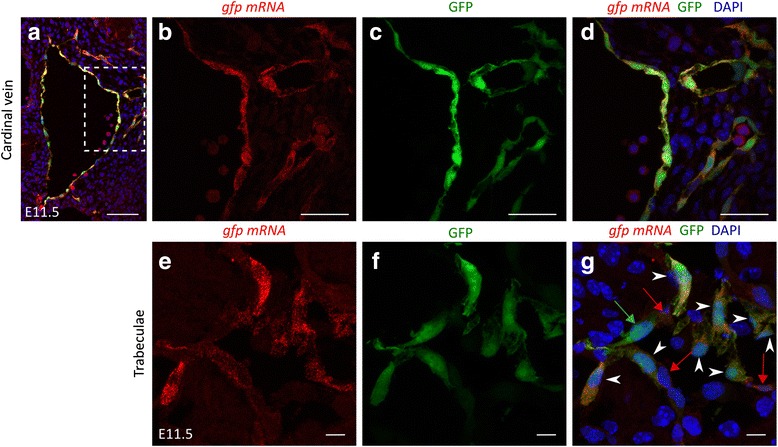
The GFP reporter protein faithfully recapitulates the transcriptional activation of the *BRE*::*gfp* transgene. In situ hybridisation for *gfp* mRNA (*red*) and direct GFP fluorescence (*green*) on cryosections through the cardinal vein (**a**–**d**) and the ventricular trabeculae (**e**–**g**) at E11.5. DAPI is used to stain nuclei. The boxed area in (**a**) is enlarged in panels (**b**–**d**). The corresponding low magnification view of the trabeculae in the ventricle is not provided because of insufficient intensity of the ISH signal. *White arrowheads* indicate ECs double positive for *gfp* mRNA and direct GFP fluorescence. The *red* and *green* *arrows* depict ECs only positive for *gfp* mRNA or GFP fluorescence respectively. Scale bars: 100 μm (**a**); 50 μm (**b**–**d**); 10 μm (**e**–**g**)

To study BMP transcriptional activity in vessel development, we used the previously generated *BRE*::*gfp* reporter strain [25]. We took advantage of the unstable nature of *gfp* mRNA, the sensitivity and single cell resolution of in situ hybridisation (ISH) on the one hand and the direct co-observation of GFP fluorescence on the other hand to validate whether GFP protein localisation reflects well the *BRE*::*gfp* transcriptional activity in endothelium. We show that there is a near to absolute correlation between the *gfp* mRNA expression and direct GFP fluorescence localisation in vascular beds like e.g. the cardinal vein (Fig. 1a–d). In other endothelial linings such as in the heart ventricles there is a good overlap between mRNA and protein, yet a fraction of the cells express only *gfp* mRNA or only GFP protein. This is indicative of onset of transcriptional activity while GFP protein translation and maturation is still taking off in the former cells, while transcriptional activity has already terminated but GFP protein is still present in the latter cells (Fig. 1e–g). This pattern suggests the dynamic turning “on” and “off” of transcriptional activity. Overall, we conclude that GFP protein patterns report with fidelity *BRE*::*gfp* transcriptional activity patterns.

The GFP localisation pattern is mostly a subdomain of the pSMAD1/5/9 pattern (Additional file 2: Figure S1A-B) as previously reported [31], which is compatible with BMP-SMAD non-transcriptional and morphogen functions as discussed in the background section of this paper. The specificity of the anti-pSMAD1/5/9 antibody was validated in endothelium-specific *Smad1*;*Smad5* double knockout embryos (Additional file 2: Figure S1D-E). However, in some endothelial beds like e.g. in the cardinal vein in E11.5 embryos, GFP localisation hardly overlapped with pSMAD1/5/9 localisation (Additional file 2: Figure S1C), suggesting terminated or undetectable pSMAD1/5/9 signalling or transcriptional activation of the *BRE*:*gfp* transgene by other factors than BMP signalling.

### *BRE*::*gfp* transcriptional activity is present in a mosaic pattern during embryonic angiogenesis

To closely examine *BRE*::*gfp* transcriptional activity in the rapidly expanding vascular plexus, we analysed embryonic day (E) 9.5 (22 somites) and E10 (30 somites) *BRE*::*gfp* mouse embryos. Tip and stalk cell formation as well as anastomosis during sprouting angiogenesis can then be investigated. In the roof of the hindbrain sprouts are formed from the perineural vascular plexus at opposite lateral sides of the embryo that then anastomose medially in a caudal fashion from the level of the otic vesicles onwards [37].

**Fig. 2:**
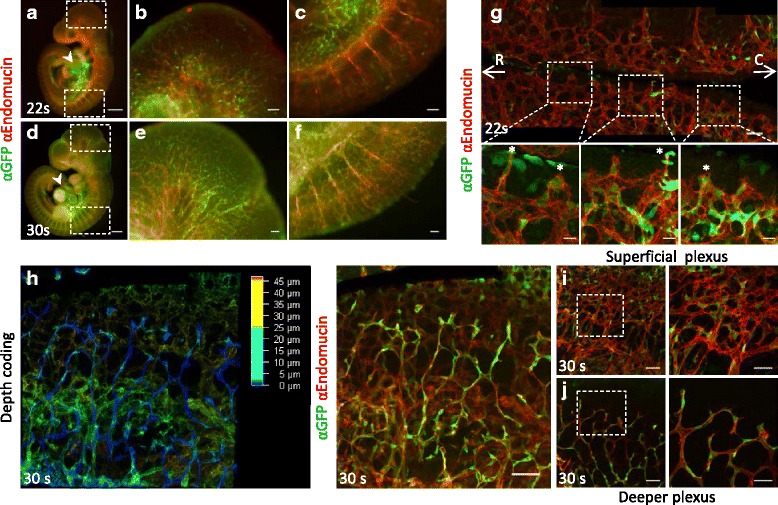
Mosaic *BRE*::*gfp* transcriptional activity in midgestation mouse hindbrain vasculature. Immunodetection of Endomucin and GFP in *BRE*::*gfp* embryos of 22 s (**a**–**c**) and 30s (**d**–**f**). *Arrowheads* indicate the heart region. Boxed areas represent the head region (**b**, **e**) and the intersomitic vessels (**c**, **f**). **g** Overview of a 22 s dorsal vascular plexus (Endomucin and GFP) in a flat-mounted hindbrain roof. *Arrows* indicate direction of rostral (R) to the left, caudal (C) to the right. Boxed areas (*bottom panels*) show sprouts at the midline. *Asterisks* depict GFP-positive tip cells. **h** Depth coding of a 30s dorsal vascular plexus and the original picture (*right panel*). *Blue* and *green* colours represent more ventral and dorsal tissues respectively. The vascular plexus at 30s consists of a superficial (**i**) and deeper plexus (**j**). Boxed areas are enlarged in the *right panels*. Scale bars: 500 μm (**a**; **d**); 100 μm (**b**–**c**; **e**–**f**); 75 μm (**g**, top; **h**; **i**–**j**, *left panels*); 50 μm (**i**–**j**, *right panels*); 25 μm (**g**, bottom)

Whole mount immunostainings of *BRE*::*gfp* embryos showed that GFP was mainly present in and around the heart region at 22 somites (s), with little *BRE*::*gfp* transcriptional activity in the Endomucin-positive blood vessels (Fig. 2a–c). However, in 30s embryos a scattered GFP pattern co-localised particularly within the main vessels of e.g. the head and the intersomitic vessels (Fig. 2d–f). The hindbrain roof was excised from these embryos and the dorsal vascular plexus was flat-mounted (Fig. 2g, top panel) [37]. In the two-dimensional vascular plexus at 22 s *BRE*::*gfp* transcriptional activity occurs in a scattered or mosaic pattern throughout the plexus, which is in accordance with previous observations [37]. Remarkably, some tip cells were found to express low levels of GFP while the mosaic pattern in non-tip cells was more intense throughout the plexus (Fig. 2g, bottom panels). This is in contrast with earlier observations where GFP was not detected in tip cells [37], which likely reflects higher affinity of the current chicken polyclonal anti-GFP antibody than the previously used mouse monoclonal anti-GFP antibody. At E9.75 the vessels from the superficial vascular plexus start to invade the hindbrain, thus forming a multi-layered vascular network [38]. Depth coding of the dorsal hindbrain region at 30s revealed the superficial and deeper plexus (Fig. 2h). The latter had slender vessels with slim tip cells, whereas the superficial plexus had thicker honeycomb-like vessels and broader tip cells, as seen at 22 s (Fig. 2g–j). The GFP localisation pattern in both plexuses was mosaic and comparable to the plexus of the 22 s embryo, yet, the deeper plexus seemed to be enriched in GFP-positive ECs (Fig. 2j) compared to the superficial plexus (Fig. 2i). Additionally, a string of GFP-positive non-endothelial cells was observed in the midline at both stages. This correlates with the presence of BMP6 and BMP7 in the dorsal midline at E9.5-E10.5 [39, 40]. In general, the two distinct dorsal vascular plexuses demonstrate heterogeneity in BMP-SMAD signalling in an angiogenic vascular bed.

### Spatio-temporal changes in *BRE*::*gfp* activity during retinal angiogenesis

The retina is commonly used to investigate postnatal blood vessel development. The primary plexus develops from the optic nerve towards the peripheral margin. Around postnatal day (P) 5 the primary vascular plexus invades the deeper retinal layers perpendicularly whereafter the outer plexus forms again radially. Different aspects of vessel formation can be studied because vascular sprouting happens at the periphery while remodelling occurs simultaneously in the centre [41, 42].

**Fig. 3:**
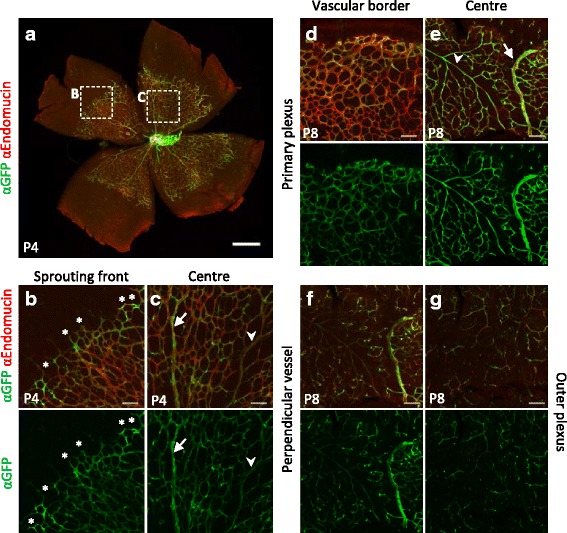
Different *BRE*::*gfp* transcriptional activity patterns in the postnatal vasculature. **a** Overview of a P4 retina with immunodetection of Endomucin and GFP. Boxed areas show the sprouting front (**b**) and the centre (**c**) of the retinal plexus. Single staining for GFP is shown in the *bottom panels*. *Asterisks* depict GFP-positive tip cells, the *arrows* point to the veins, while the *arrowheads* show the arteries. **d**–**g** The retina at P8, immunostained for Endomucin and GFP, has a multi-layered vascular plexus consisting of the vascular border (**d**) and centre (**e**) of the primary plexus, the perpendicular vessels (**f**) and outer plexus (**g**). Single GFP staining is shown in the *lower panels*. Scale bars: 500 μm (**a**); 75 μm (**b–g**)

The GFP localisation pattern was diverse but stereotypic throughout the retinal stages investigated and the GFP levels decreased over time. At P4, the vasculature has sprouted halfway across the retina (Fig. 3a). The sprouting front displayed relatively strong GFP signals compared to the central plexus (Fig. 3b–c). Many tip cells at the sprouting front as well as the arteries and veins in the centre were GFP-positive, whereas the intermediate capillary bed displayed a more mosaic GFP distribution (Fig. 3b–c). Moreover, arteries seemed weaker GFP-positive compared to the strong GFP-positive veins (Fig. 3c). At P8 the sprouting front developed into a vascular border. Comparable to P4, GFP-positive arteries and veins were observed, along with a mosaic distribution of GFP in the capillaries (Fig. 3d–e). At this stage the matured vessels in the centre have sprouted into the retina to form the perpendicular vessels and the outer plexus. Approximately half of the perpendicular vessels seemed GFP-positive, though weaker than the veins of the primary plexus (Fig. 3e–f). Moreover, the less ramified outer plexus also displayed a mosaic GFP pattern (Fig. 3g). Overall, fewer GFP-positive cells were present in the different plexuses at P10, although the mosaic distribution was maintained in the primary and outer plexus (Additional file 3: Figure S2). These data indicate a dynamic nature of BMP-SMAD signalling over time.

### GFP localisation patterns are dynamic in the developing heart

**Fig. 4:**
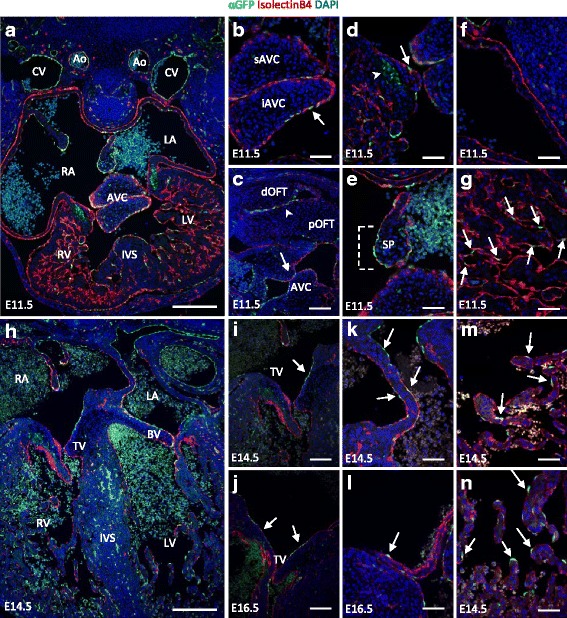
Dynamic *BRE*::*gfp* transcriptional activity in the developing heart. Paraffin embedded hearts are sectioned transversally, unless otherwise specified, and (immuno) stained for the endothelial marker IsolectinB4 and GFP. DAPI is used to stain nuclei. **a** Overview of an E11.5 heart. **b** Section of the superior and inferior atrioventricular canal cushions at E11.5. **c** E11.5 sagittal section of AVC (*arrow*) and OFT cushions (*arrowhead*). **d** E11.5 lateral cushion with GFP-positive endocardial cells (*arrow*) and myocardium (*arrowhead*). **e** At E11.5 the septum primum displays GFP-positive ECs on the endothelial cap (*bracket*). The atrium is GFP-negative at E11.5 (**f**) while the ventricular trabeculae (**g**) have GFP-positive ECs (*arrows*). **h** Overview of an E14.5 heart. The tricuspid valve shows GFP-positive cells at the atrial side (*arrows*) at E14.5 (**i**) and E16.5 (**j**). GFP signals (*arrows*) in the intra-atrial septum at E14.5 (**k**) and E16.5 (**l**). Some endocardial cells covering the atrial (**m**) and ventricular trabeculae (**n**) are GFP-positive (*arrows*) at E14.5. Ao: aorta; AVC: atrioventricular canal; iAVC: inferior AVC cushion; sAVC: superior AVC cushion; BV: bicuspid valve; CV: cardinal vein; dOFT: distal outflow tract; IVS: inter-ventricular septum; LA: left atrium; LV: left ventricle; pOFT: proximal outflow tract; RA: right atrium; RV: right ventricle; SP: septum primum; TV: tricuspid valve. Scale bars: 200 μm (**a**;**h**); 100 μm (**c**, **i**-**j**); 50 μm (**b**, **d**-**g**, **k**-**n**)

Given the well-known functions of BMPs in heart development and the presence of *BRE*::*gfp* activity during early heart development and valve formation [25], we zoomed in deeper on the spatio-temporal patterns of *BRE*::*gfp* transcriptional activity in endocardium at E9.5-E12.5, E14.5 and E16.5 and observed remarkable regional differences over time. At E8.0 the initial heart tube starts to loop and progressively forms the four chambered heart [43, 44]. The heart tube comprises an inner endothelial layer and an outer myocardial layer separated by extracellular matrix or cardiac jelly [45]. The pro-epicardial organ progressively covers the myocardial layer with epicardium. Overall, relatively few GFP-positive cells were observed in tissue sections of the developing heart (Fig. 4), which is consistent with former results [25]. Nonetheless, GFP patterns were robust and reproducible in endocardial subregions, and changed over time (Additional file 4: Table S2).

Cardiac cushions are the primordia of the valves and most septa in the developing heart. They are formed in the atrioventricular canal (AVC) which separates the atria from the ventricles and the outflow tract (OFT) which bridges the ventricles with the aortic sac [43–45]. At respectively E9.5 and E10.5 the endocardial cells of the AVC cushions and proximal OFT cushions delaminate, undergo endothelial-to-mesenchymal transition (EndMT) and invade the cardiac jelly [43–45]. In contrast, the distal OFT cushions become mainly populated by neural crest cell derived mesenchymal cells from E10.5 onwards [46].

The endocardial cells of the superior and inferior AVC cushions displayed a mosaic GFP localisation pattern until E11.5 (Fig. 4a–c). From E12.5 onwards, patches of GFP-positive ECs were restricted to the atrial side of the superior cushion. These streaks of GFP-positive ECs remained present at E14.5 and E16.5 on the medial leaflets of the tricuspid and bicuspid valve that form from these cushions (Fig. 4h–j). In contrast, only few GFP-positive ECs were present in the lateral AVC cushions from E11.5 onwards (Fig. 4d; i). Yet, at E16.5 the lateral leaflets of the tricuspid and bicuspid valve displayed, just like the medial leaflets, also patches of GFP-positive ECs (Fig. 4j). Patches of GFP-positive muscle cells were also observed in the AVC myocardium flanking the developing cushions and valves up to E14.5, though with varying GFP levels among neighbouring cells. (Fig. 4d; h–i). The ECs of the OFT cushions showed a mosaic GFP localisation pattern comparable to the AVC cushions at E11.5, although the OFT appeared slightly enriched in GFP-positive ECs (Fig. 4c). Remarkably, the mesenchymal cells that populate the AVC and OFT cushions were GFP-negative (Fig. 4a–c). In contrast to the ECs of the AVC and OFT, the ECs of the inflow tract and the endothelial cap of the septum primum showed a ubiquitous GFP localisation pattern at E11.5 (Fig. 4e and Additional file 5: Figure S3A). As development proceeds and this septum reaches the superior cushion forming the intra-atrial septum, *BRE*::*gfp* transcriptional activity decreased with only a few GFP-positive ECs still present at E14.5 and E16.5 (Fig. 4k–l).

Only occasionally a GFP-positive cell was detected in atrial endothelium at E11.5 (Fig. 4f), while the ventricles showed a mosaic GFP pattern in the ECs covering the trabeculae throughout the stages analysed (Fig. 1e–g, Fig. 4g). Interestingly, a mosaic pattern also emerged in the atrial endothelium coinciding with initiation of trabeculation from E12.5 onwards (Fig. 4m–n). Moreover, the ECs of the aortic and pulmonary valve leaflets also displayed a mosaic GFP pattern at E14.5 (Additional file 5: Figure S3B–C).

### Levels of *BRE*::*gfp* activity differ in embryonic and postnatal blood and lymphatic vessels

Little information is available on BMP-SMAD signalling in different lymphatic beds. At E9.75 the first LECs differentiate from venous ECs in the cardinal vein [1]. These LECs bud of, migrate and assemble into lymphatic sacs by E11.5, which will remodel into a functional lymphatic network. We found interesting spatio-temporal differences in *BRE*::*gfp* transcriptional activity during lymphangiogenesis. In the embryo most blood and lymphatic vessels have a widespread GFP localisation pattern, while in postnatal tissues like the mesentery, intestinal villi and the ear skin many blood vessels appeared to have reduced GFP signals and the lymphatic vessels had discrete and unique GFP localisation patterns.

**Fig. 5:**
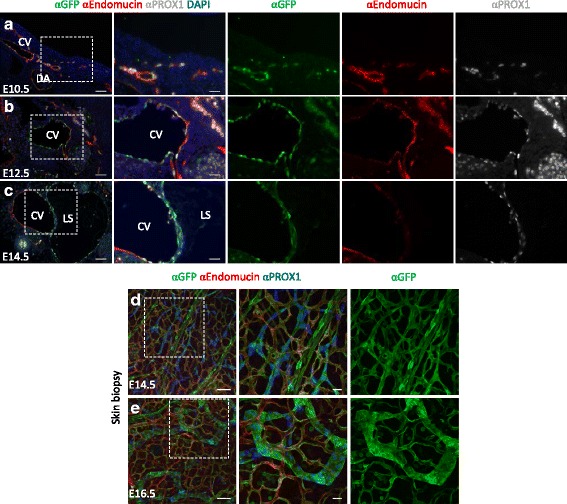
Spatio-temporal changes in *BRE*::*gfp* transcriptional activity in blood and lymphatic vessels. **a**–**c** Transverse sections at the level of the neck of E10.5 (**a**), E12.5 (**b**) and E14.5 (**c**) *BRE*::*gfp* embryos with immunodetection of GFP, the blood vessel marker Endomucin and the lymphatic vessel marker PROX1. DAPI is used to stain nuclei. Boxed areas are enlarged in the *right* panels, with single GFP, Endomucin and PROX1 staining next to it. Skin biopsies of E14.5 (**d**) and E16.5 (**e**) *BRE*::*gfp* embryos with immunodetection of GFP, Endomucin and PROX1. Boxed areas are shown in the *middle* (and *right*) panels with single staining for GFP in the *right* panel. CV: cardinal vein; DA: dorsal aorta; LS: lymphatic sac. Scale bars: 75 μm (**d**–**e**, *left* panels); 50 μm (**a**–**c**, *left* panels; **d**–**e**, *right* panels); 25 μm (**a**–**c**, *right* panels)

In general, the blood vessels, including the cardinal vein, and lymphatic vessels displayed a continuous GFP localisation pattern at E11.5-E14.5 (Fig. 1a-d, Fig. 4a, Additional file 6: Figure S4A-B). However, the aorta showed a mosaic GFP localisation pattern (Fig. 4a). Interestingly, nearly all PROX1- positive LECs budding from the cardinal vein were GFP-positive, yet still weakly positive for the blood vessel marker Endomucin in E10.5 embryos (Fig. 5a). Likewise, at E12.5 some GFP-positive ECs were PROX1- and Endomucin-positive (Fig. 5b), yet in few sections at E14.5 the GFP-positive cardinal vein adjacent to the lymphatic sac was Endomucin-positive on the medial side, while the lateral side, closest to the lymphatic sac, was PROX1-positive (Fig. 5c). Blood and lymphatic vessels in dorsal skin biopsies were also ubiquitously GFP-positive at E14.5 and E16.5 (Fig. 5d–e and Additional file 6: Figure S4C). However, the GFP signal appeared more uniform in the blood vessels, whereas the LECs showed different levels of GFP among neighbouring cells (Fig. 5d–e). Other GFP-positive cord-like structures were observed at E14.5 and E16.5 in dorsal skin biopsies, but these were excluded as vessels because they were Collagen Type IV-negative (Additional file 7: Figure S5). Similar structures have been described as Schwann cells of sensory nerves [47].

Since the lymphatic network is still expanding after birth, we investigated the postnatal mesentery, intestines and ear skin. Different types of lymphatic vessels occur in each of these tissues. The mesentery contains collecting vessels, while lacteals resorb lipids from the intestines and lymphatic capillaries drain lymph from ear tissue.

**Fig. 6:**
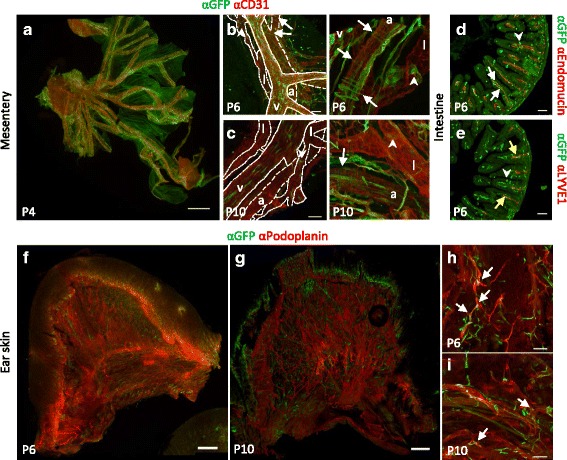
*BRE*::*gfp* transcriptional activity in postnatal blood and lymphatic vessels. **a** Stitch of a P4 mesentery with immunodetection of GFP and the pan-endothelial marker CD31. Arteries, veins and collecting lymphatic vessels in a P6 (**b**) and P10 (**c**) mesentery are depicted. *Arrows* point to GFP-positive peri-endothelial cells. *Arrowheads* show GFP-positive ECs in the vicinity of a valve. Sections of P6 intestines with immunodetection of GFP and Endomucin (**d**) or the lymphatic vessel marker LYVE1 (**e**). *White arrows* point to some GFP-positive cells in a capillary, *yellow arrows* show GFP-positive LECs in a lacteal. *Arrowheads* show some GFP-positive goblet cells. Stitch of P6 (**f**) and P10 (**g**) dorsal ear skin with immunodetection of GFP and the lymphatic marker Podoplanin. GFP-positive LECs at the branch points are depicted by *arrows* at P6 (**h**) and P10 (**i**). A: artery; L: lymphatic vessel; V: vein. Scale bars: 1 mm (**a**); 500 μm (**f**–**g**); 75 μm (**b**–**e**; **h**–**i**)

Interestingly, many venous ECs appeared GFP-positive in P4 and P6 mesentery (Fig. 6a–b), whereas only few GFP-positive arterial ECs were observed (Fig. 6a–b) with also some GFP-positive peri-endothelial cells covering the arteries (Fig. 6b). The developing lymphatic vessels were predominantly GFP-negative, although *BRE*::*gfp* transcriptional activity was specifically present in some valve forming regions (Fig. 6b). At P10, the arterial ECs and peri-endothelial cells appeared GFP-negative and also fewer venous ECs were GFP-positive, whereas the lymphatic vessels still showed GFP in the valve regions (Fig. 6c). Remarkably, the vessels had reduced *BRE*::*gfp* transcriptional activity over time from P4 to P10 (Fig. 6a–c).

Most intestinal villi comprise a LYVE1-positive lymphatic vessel, called the lacteal, which is surrounded by an Endomucin-positive blood capillary. Only few ECs of the blood capillaries and lacteals were GFP-positive (Fig. 6d–e). Other non-endothelial cells, such as goblet cells, were also GFP-positive in the villus (Fig. 6d–e). In the ear skin *BRE*::*gfp* transcriptional activity was absent from the lymphatic capillary bed at P6 and P10, with the exception of a single GFP-positive LEC at some branch points (Fig. 6f–i).

### *BRE*::*gfp* transcriptional activity does not correlate with proliferation

**Fig. 7:**
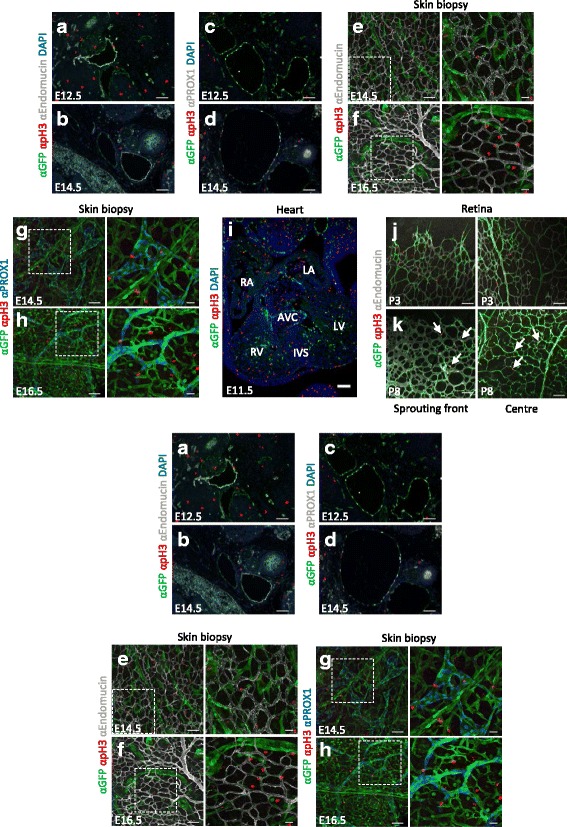
*BRE*::*gfp* transcriptionally active ECs are rarely proliferative. Transverse sections of E12.5 (**a**, **c**) and E14.5 (**b**, **d**) *BRE*::*gfp* mouse embryos with immunodetection of the proliferation marker (pH3), GFP and Endomucin (**a**–**b**) or PROX1 (**c**–**d**). Dorsal skin biopsies obtained from E14.5 (**e**, **g**) and E16.5 (**f**, **h**) *BRE*::*gfp* embryos show pH3, GFP and Endomucin (**e**–**f**) or PROX1 (**g**–**h**) staining. Boxed areas are enlarged in the *right panels*. **i** Overview of an E11.5 heart with immunodetection of GFP and pH3. DAPI is used to stain nuclei. The sprouting front (*left panels*) and centre (*right panels*) of P3 (**j**) and P8 (**k**) retinas with immunodetection of GFP, Endomucin and pH3. *Arrows* point to pH3-positive ECs. AVC: atrioventricular canal; IVS: inter-ventricular septum; LA: left atrium; LV; left ventricle; RA: right atrium; RV: right ventricle. Scale bars: 100 μm (**i**); 75 μm (**e**–**h**, *left panels*; **j**–**k**); 50 μm; 25 μm (*right panels*)

Inhibitors of differentiation or IDs are helix-loop-helix proteins that interact with and inhibit basic helix-loop-helix transcription factors. Through induction of these IDs, the BMP pathway promotes EC migration and tube formation [48]. This led to the hypothesis that GFP-positive ECs would be proliferative. Several embryonic and postnatal tissues were investigated for co-localisation of GFP and phospho histone 3 (pH3) in the blood and lymphatic system. PH3 is only present in the M-phase of the cell cycle [49]. There was rarely overlap between pH3 and GFP-positive blood or lymphatic ECs in the neck region at E12.5 and E14.5 (Fig. 7a–d) or in dorsal skin biopsies from E14.5 and E16.5 embryos (Fig. 7e–h). When pH3 seemed to be present in a GFP-positive EC, the proliferating cell was usually not in the same focal plane as the EC (Additional file 8: Figure S6A), or showed mainly low to no levels of GFP (data not shown). In addition, skin biopsies of E14.5 and E16.5 embryos were immunostained for Ki67 which marks all active phases of the cell cycle. Proliferation decreased between E14.5 and E16.5, but even though many non-endothelial cells were proliferative only few GFP-positive ECs and LECs were Ki67-positive (Additional file 8: Figure S6B-C). Also in the heart there was almost no co-localisation observed between pH3 and GFP (Fig. 7i). Furthermore, ECs of the sprouting front and the centre of the retina were not pH3-positive at P3, however, at this stage non-endothelial cells were more proliferative at the sprouting front than at the centre (Fig. 7j). Overall, proliferation decreased by P8 but shifted towards the ECs, as now pH3-positive ECs could be observed (Fig. 7k). Some of these proliferating cells seemed more GFP-positive than others. This is an intriguing difference in co-localisation pattern which suggests another context dependent role for BMP-SMAD signalling.

## Discussion

Spatio-temporal information on output of important signalling pathways in the vasculature may help to increase our understanding of how mutations in components of the same pathway can cause organ-specific vascular disorders and provide a window of opportunity for designing disease-specific therapy. In the past decade, many BRE-reporters have been generated in zebrafish and mice [25–30]. The *BRE*::*gfp* reporter used in this study is not the most sensitive, as some other BMP reporters show broader patterns of transcriptional activity. However, this precisely allows to zoom in on selective processes and dose-dependent BMP actions. Our study shows that GFP patterns in endothelium faithfully report transcriptional activation of the *BRE*::*gfp* transgene, and are remarkably robust. However the BMP-SMAD transcriptional output is highly dynamic in time and space, in the different cardiovascular and lymphatic beds of *BRE*::*gfp* mice.

Mosaic GFP localisation patterns were observed in different regions of the developing vascular tree and heart. It was found in the dorsal vascular plexuses of midgestation mouse embryos and in the capillary bed of P4-P10 retinas. In addition, at E9.5-E11.5 the endocardial cells of the AVC and OFT cushions and those lining the atrial and ventricular trabeculae also displayed a mosaic GFP pattern (Additional file 5: Table S2). Such a mosaic pattern of transcriptional activity suggests a role for BMP-SMAD in EC plasticity and micro-heterogeneity. The multi-layered vascular network of the dorsal hindbrain and retina develop in a similar fashion, with the deeper plexus of the dorsal hindbrain resembling morphologically more the capillary network in the primary plexus of the retina [38]. Previously, we showed that the *BRE*::*gfp* pattern singled out stalk cell competent cells in the dorsal vascular plexus that were undergoing BMP and Notch co-signalling and that loss of BMP-SMAD signalling in endothelium resulted in a stalk cell defect [37]. We also observed weak GFP-positive tip cells in the dorsal hindbrain and retinal plexuses. These tip cells might have been former stalk cells that have taken over the tip cell position [50], with traces of non-degraded GFP. Alternatively, BMP6 and BMP7 synthesized by cells at the midline [39, 40] may function as pro-angiogenic guidance cues that trigger an alternative BMP-SMAD signalling pathway in the tip cells. Circulatory BMP9 is likely to promote stalk cell competence through activating the mosaic transcriptional activity observed in the rest of the dorsal vascular plexus. In the retina, BMP9 and BMP10 are important for postnatal vascular remodelling [51]. Remarkably, BMP10 was unable to induce BRE activity in vitro, suggesting that the GFP signals in the centre of the retina, where vessel maturation and remodelling occurs, were the result of BMP9 signalling. However, also BMP2, 4, 6 and 7 have been shown to play significant roles in retinal neurogenesis and vascularisation [52]. Retinal vascularisation is preceded and stimulated by the development of a vast network of neuronal cells [53], the latter also depending on BMP-SMAD signalling [52, 54]. The retinal ECs reciprocally promote differentiation of the neuronal plexus [53]. Our data do not allow to distinguish the precise source and type of BMP signal, yet, BMP-SMAD transcriptional activity seems more imperative at the sprouting front than in the centre where the vascular plexus is maturing.

Many BMPs have been reported to regulate cardiac cushion development [43, 45]. In the AVC cushions, BMP2 stimulates ECs to undergo EndMT [55], while BMP4 is important in the OFT for proliferation and growth of endocardial cushions rather than EndMT [56]. Studies with knockout mice reveal that BMP2, ALK2, ALK3, BMPR2, SMAD4 and SMAD6 are important for the development of the AVC cushions and to a lesser extent OFT cushions [55, 57–64]. Our study shows that BMP signalling induces mosaic transcriptional activity in cushion endocardium, likely to maintain an intact cushion epithelium while a few cells can undergo EndMT. Whether the GFP-positive cells or rather their neighbours are subsequently triggered to undergo EndMT remains to be elucidated. Taken together, in cushion endocardium and in angiogenic endothelium, the mosaic-perhaps stochastic-transcriptional BMP-SMAD activity seems to serve as a source of phenotypic diversity. The exquisite fine-tuning of the BMP pathway, which also involves negative feedback mechanisms, may also generate switch modes of activation states. Whether the mosaicism in BMP-SMAD transcriptional activity is static or dynamic, with *BRE*::*gfp* activity switching between ‘on’ and ‘off’ states, cannot be addressed directly in our model due to limitations in the resolution of real-time intravital microscopy, combined with the need for potentially long windows of observation. Dynamic mosaicism in expression has recently been demonstrated for von Willebrand factor (VWF), and also in vitro for ESM1 and ephrin-B2, in some but not all vascular beds. This appears to be a phenotype switching strategy for adaptive homeostasis [65].

Remarkably, the endocardial cells of the atria turned on mosaic *BRE*::*gfp* activity several days later than the ventricular endocardial cells. Interestingly, this delayed activation correlated with the delayed onset of *BMP10* expression [66] and initiation of trabeculation in atrial myocardium at E12.5 compared to the onset of the same process in ventricular myocardium already at E9.5. BMP10 is a well-known regulator of cardiac trabeculation and/or compaction [67]. Trabeculation defects are also observed in endothelium-specific *Smad4* KO and *Smad1*/*Smad5* double KO embryos [37, 68]. Our data suggest that not all ECs are equally involved in this process. It would be interesting to evaluate whether and how expansion from a mosaic to a continuous BMP-SMAD transcriptional activity pattern in ventricular and atrial endocardium would impact trabeculation or provide (fitness) advantages.

Remarkably, during embryonic development the lymphatic vessels showed widespread *BRE*::*gfp* transcriptional activity, yet in pups GFP-positive ECs were restricted to the valve forming regions of collecting lymphatic vessels in the mesentery. This is in agreement with the role of BMP9 in lymphatic valve development [69]. In the lymphatic capillary bed of the ear skin, an occasional GFP-positive cell would localise at branch points. Furthermore, GFP-positive endocardial cells were observed on the atrial side of the tricuspid and bicuspid heart valve leaflets, but also in the inflow tract. All these patterns correspond with endothelium undergoing fluid shear stress, which can induce BMP-SMAD signalling. Hemodynamic alterations have been reported to induce BMP4 and activate SMAD1/5 in the aorta [18, 70, 71], and to mediate arteriogenesis [19].

Mature ECs are characterized by a slow proliferation rate. For example, in adult ear skin only 0.2 % of the LECs are reported to be Ki67-positive, whereas approximately 30 % of LECs are Ki67-positive in embryonic skin at E16.5-E17.5 [72]. The role of BMP signalling in EC proliferation is thought to be highly context dependent [73]. Because *BRE*::*gfp* signals peaked around midgestation and progressively decreased in postnatal stages in the vascular tree, we reasoned that correlations between BMP-SMAD transcriptional output and proliferation may become apparent in specific vascular beds. Remarkably, we found that proliferating pH3-positive ECs were almost invariably GFP-negative in the different vascular beds analysed, except in the P10 retina where more often double positive cells were observed.

A recurrent theme was - like in zebrafish embryos [74] - that the abundance of GFP-positive ECs was higher in veins than in arteries in several embryonic and postnatal tissues. For instance, the ECs in the cardinal vein showed a continuous GFP localisation pattern, whereas the aorta had a mosaic GFP pattern. In the mesentery almost no GFP-positive ECs were observed in the arteries, yet some of its peri-endothelial cells were GFP-positive. In contrast, many ECs of the veins were GFP-positive. ALK1, Endoglin and BMPR2 play a role in the establishment and maintenance of mural cell coverage on mature vessels [75, 76], primarily in arteries. Mutations in BMPR2 lead to PAH which is characterized by abnormal proliferation of ECs and smooth muscle cells (SMCs) in arterioles [73, 77], whereas a deletion of BMPR2 leads to insufficient recruitment and decreases *PDGFRβ* expression in mural cells [77]. BMP2/BMPR2 signalling negatively regulates PDGFBB induced proliferation of pulmonary arterial SMCs in a pSMAD1/5/9 independent manner [77]. It is likely that veins express more BMP2 and hence limit the number of SMC coverage, but also the differences in shear stress in both vessel types may underlie the above differences.

## Conclusion

We experienced that the *BRE*::*gfp* reporter is an exquisitely useful tool to get grip on the complex BMP-SMAD transcriptional signalling contexts in vivo. The GFP signals are robust, reproducible and highly regionalised; they correlate well with known areas of BMP signalling in the endothelium and reveal new microdomains of BMP signalling. Our study underscores the regionalised and heterogeneous nature of BMP signalling in the circulatory and lymphatic vasculature of embryos and pups, with striking shifts in transcriptional output over time in different endothelium types. This study highlights that extrapolation of results obtained in one vascular bed to another, or generalisation, should be done with extreme care. Examining other BMP signalling reporters and intercrossing them can likely shed light on yet other facets of the complex BMP-SMAD signalling output. Knowledge on differential signalling output is highly valuable to better understand the ontogeny of BMP-linked diseases and may lead to improved disease-tailored therapies.

## Additional files

Additional file 1: Table S1. List of primary antibodies and glycoprotein stainings. Biotin amplification was only required for PROX1 detection. The same antibody concentration was used for whole mount analysis, paraffin sections and cryosections, unless otherwise indicated. (PDF 226 kb)
Additional file 2: Figure S1. The BRE::gfp reporter is transcriptionally active in a subdomain of the pSMAD1/5/9-positive endothelial cells. (A–C) Immunodetection of SMAD1/5/9 and GFP in the AVC and OFT at E9.5 (A), the ventricular trabeculae (B) and the cardinal vein (C) at E11.5. DAPI is used to stain nuclei. Specificity of the pSMAD1/5/9 staining was confirmed in control embryos (D) and endothelium-specific *Smad1*; *Smad5* double knockout embryos (Tie2cre^+/0;^ Smad1^fl/fl^; Smad5^fl/fl^) (E). Embryos were analyzed at E8.5 to circumvent the embryonic lethality of mutant embryos at E9.5. The pSMAD1/5/9 levels were specifically reduced in endothelium of the EC-specific *Smad1*; *Smad5* double knockouts, while levels remained unchanged in the non-endothelial cells. The myocardium was visualized by an anti-MF20 staining. *Arrows* indicate pSMAD1/5/9 deficient endothelium (E). At: atrium; AVC: atrioventricular canal; CV: cardinal vein; OFT: outflow tract. Scale bars: 100 μm (A–B;D–E); 50 μm (C). (PDF 2307 kb)
Additional file 3: Figure S2.
*BRE*::*gfp* localisation patterns in the P10 retina. (A–D) Immunodetection of Endomucin and GFP in the retina at P10. The retina has a multi-layered vasculature that consist of the vascular border (A) and centre (B) of the primary plexus, the perpendicular vessels (C) and outer plexus (D). Single staining for GFP is shown in the *lower panels*. Scale bars: 75 μm. (PDF 2844 kb)
Additional file 4: Table S2. GFP localisation patterns in embryonic hearts with emphasis on *BRE*::*gfp* activity in endocardial cells. The number of hearts analysed is indicated in the top row. Results are consistent for all hearts analysed, unless specifically indicated. (C) Continuous (100 %); (U) Ubiquitous (>70 %); (M) Mosaic (10–70 %); (O) Occasional cell (<10 %); (P) Patches; (ND) undetectable; N/A not applicable (PDF 215 kb)
Additional file 5: Figure S3.
*BRE*::*gfp* transcriptional activity in the outflow tract and aortic and pulmonary valves. Transverse sections of E11.5 embryos with (immuno) detection of Isolectin B4 and GFP. DAPI is used to stain nuclei. (A) Ubiquitous GFP signal in the inflow tract valve, and mosaic GFP localisation in the aortic (B) and pulmonary (C) valve. Scale bars: 50 μm (PDF 181 kb)
Additional file 6: Figure S4.
*BRE*::*gfp* transcriptional activity is similar in blood and lymphatic vessels. Transverse sections through the neck of E12.5 (A) and E14.5 (B) *BRE*::*gfp* embryos immunostained for GFP, Endomucin, PROX1 and DAPI. Boxed areas are enlarged in the *right panels* with the single GFP staining next to it. (C) Schematic overview shows the dorsal skin area taken from an E14.5 embryo. Scale bars: 50 μm (A-B, left panels); 25 μm (A–B, right panels). (PDF 136 kb)
Additional file 7: Figure S5.
*BRE*::*gfp* transcriptional activity is not restricted to vessels. E14.5 skin biopsies with immunodetection of GFP, PROX1 and the extracellular matrix marker Collagen type IV. Boxed areas are enlarged in the *right panels*. *Arrows* show non-vessel structures. Scale bars: 75 μm (left panels); 25 μm (right panels). (PDF 184 kb)
Additional file 8: Figure S6.
*BRE*::*gfp* transcriptional activity does not co-localise with Ki67 in ECs. (A) Skin biopsy of an E14.5 *BRE*::*gfp* mouse with immunodetection of GFP, pH3 and PROX1. The merged stack in the *left panel* is split up in some single z-stacks to show pH3-positive cells beneath or above vessel structures (*arrows*). E14.5 (B) and E16.5 (C) skin biopsies with immunodetection of a different proliferation marker (Ki67), GFP and PROX1. Boxed areas are enlarged in the *right* panels. Ki67-positive LECs (*arrows*) and blood ECs (*arrowheads*) can be observed. Scale bars: 75 μm (B–C, left panels); 25 μm (A; B–C, right panels). (PDF 184 kb)

## Abbreviations

(e) GFP: (enhanced) green fluorescent protein
A: Artery
Ao: Aorta
At: Atrium
AVC: Atrioventricular canal
BMP: Bone morphogenetic protein
BRE: BMP response element
BSA: Bovine serum albumin
BV: Bicuspid valve
CV: Cardinal vein
DA: Dorsal aorta
DEPC: Diethylpyrocarbonate
dOFT: Distal outflow tract
E: Embryonic day
EC: Endothelial cell
EndMT: Endothelial-to-mesenchymal transition
FISH: Fluorescent in situ hybridisation
HHT: Hereditary hemorrhagic telangiectasia
iAVC: Inferior atrioventricular canal cushion
ID: Inhibitor of differentiation
ISH: *In situ* hybridisation
IVS: Inter-ventricular septum
L: Lymphatic vessel
LA: Left atrium
LEC: Lymphatic endothelial cell
LS: Lymphatic sac
LV: Left ventricle
OCT: Optimal cutting temperature
OFT: Outflow tract
ON: Overnight
P: Postnatal day
PAH: Pulmonary arterial hypertension
PBS: Phosphate buffered saline
PFA: Paraformaldehyde
pH3: Phospho histone 3
pOFT: Proximal outflow tract
pSMAD: Phosphorylated SMAD
RA: Right atrium
RT: Room temperature
RV: Right ventricle
S: Somites
sAVC: Superior atrioventricular canal cushion
SMC: Smooth muscle cell
SP: Septum primum
TBS: Tris buffered saline
TGFβ: Transforming growth factor beta
TV: Tricuspid valve
V: Vein
VWF: von Willebrand factor
