# Supplementary material for: BMP-SMAD signalling output is highly regionalized in cardiovascular and lymphatic endothelial networks
Source: BMC Dev Biol. 2016 Oct 10;16:34. doi: 10.1186/s12861-016-0133-x (PMC5057272; doi:10.1186/s12861-016-0133-x)
Supplement: Additional file 1: Table S1. — List of primary antibodies and glycoprotein stainings. Biotin amplification was only required for PROX1 detection. The same antibody concentration was used for whole mount analysis, paraffin sections and cryosections, unless otherwise indicated. (PDF 226 kb) [file 12861_2016_133_MOESM1_ESM.pdf]

Primary antibody name	Concentration for whole mount/paraffin/cryosections	Company	Catalogue #
**CD31**	1/100	BD Pharmingen	553370
**Collagen type IV**	1/100	AbdSerotec	2150-1470
**Endomucin**	1/100	Santa Cruz	sc65495
**GFP**	1/100; 1/500	Abcam	ab13970
**Isolectin B4**	1/100	Sigma-Aldrich	L2140-1MG
**Ki67**	1/100	Abcam	ab16667
**LYVE1**	1/100	Abcam	ab14917
**pH3**	1/100	Abcam	ab5176
**Podoplanin**	1/100	R&D systems	AF3244
**PROX1**	1/100 biotin	R&D systems	AF2727
**MF20**	1/100	R&D systems	MAB4470
**pSMAD1/5/9**	1/300	Cell Signaling	#13820
