# Supplementary figures and images for: BMP-SMAD signalling output is highly regionalized in cardiovascular and lymphatic endothelial networks
Source: BMC Dev Biol. 2016 Oct 10;16:34. doi: 10.1186/s12861-016-0133-x (PMC5057272; doi:10.1186/s12861-016-0133-x)

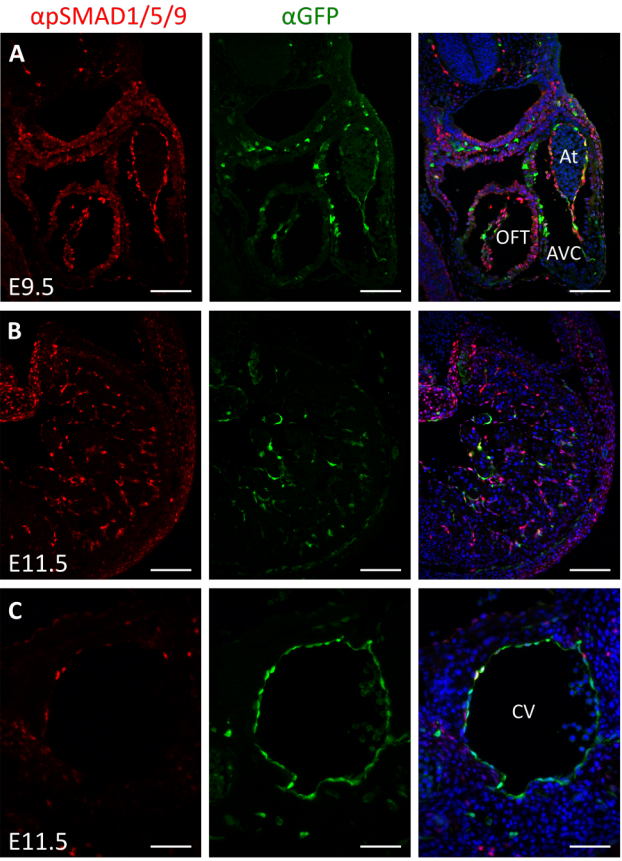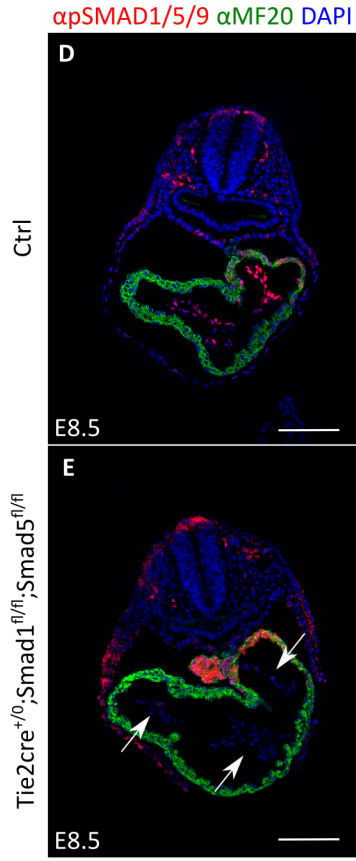

Supplement: Additional file 2: Figure S1. — The BRE::gfp reporter is transcriptionally active in a subdomain of the pSMAD1/5/9-positive endothelial cells. (A–C) Immunodetection of SMAD1/5/9 and GFP in the AVC and OFT at E9.5 (A), the ventricular trabeculae (B) and the cardinal vein (C) at E11.5. DAPI is used to stain nuclei. Specificity of the pSMAD1/5/9 staining was confirmed in control embryos (D) and endothelium-specific Smad1; Smad5 double knockout embryos (Tie2cre+/0; Smad1fl/fl; Smad5fl/fl) (E). Embryos were analyzed at E8.5 to circumvent the embryonic lethality of mutant embryos at E9.5. The pSMAD1/5/9 levels were specifically reduced in endothelium of the EC-specific Smad1; Smad5 double knockouts, while levels remained unchanged in the non-endothelial cells. The myocardium was visualized by an anti-MF20 staining. Arrows indicate pSMAD1/5/9 deficient endothelium (E). At: atrium; AVC: atrioventricular canal; CV: cardinal vein; OFT: outflow tract. Scale bars: 100 μm (A–B;D–E); 50 μm (C). (PDF 2307 kb) [file 12861_2016_133_MOESM2_ESM.pdf]

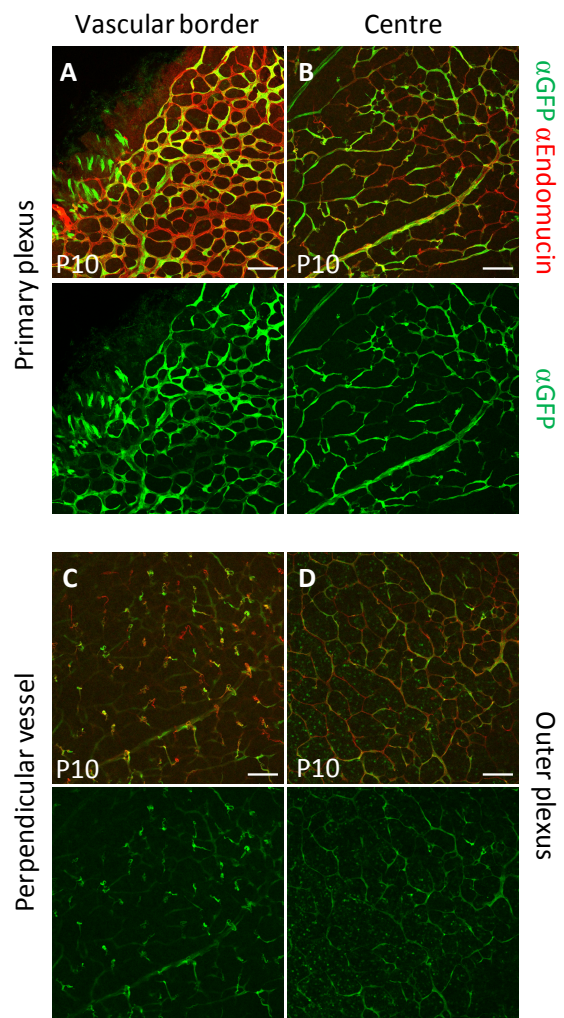

Supplement: Additional file 3: Figure S2. — BRE::gfp localisation patterns in the P10 retina. (A–D) Immunodetection of Endomucin and GFP in the retina at P10. The retina has a multi-layered vasculature that consist of the vascular border (A) and centre (B) of the primary plexus, the perpendicular vessels (C) and outer plexus (D). Single staining for GFP is shown in the lower panels. Scale bars: 75 μm. (PDF 2844 kb) [file 12861_2016_133_MOESM3_ESM.pdf]

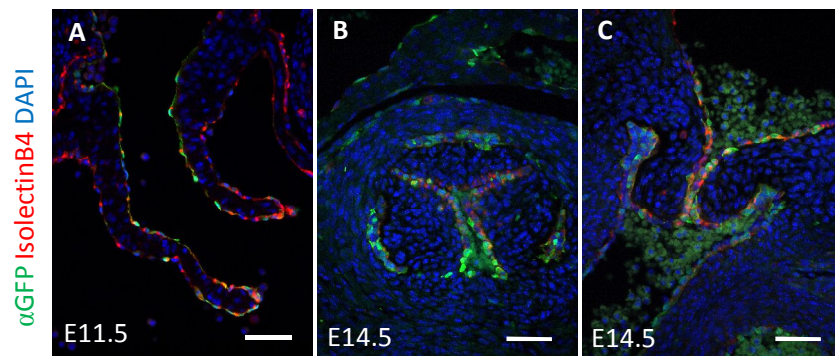

Supplement: Additional file 5: Figure S3. — BRE::gfp transcriptional activity in the outflow tract and aortic and pulmonary valves. Transverse sections of E11.5 embryos with (immuno) detection of Isolectin B4 and GFP. DAPI is used to stain nuclei. (A) Ubiquitous GFP signal in the inflow tract valve, and mosaic GFP localisation in the aortic (B) and pulmonary (C) valve. Scale bars: 50 μm (PDF 181 kb) [file 12861_2016_133_MOESM5_ESM.pdf]

$\alpha$ GFP  $\alpha$ Endomucin  $\alpha$ PROX1 DAPI

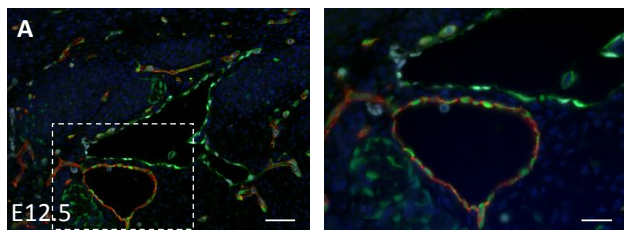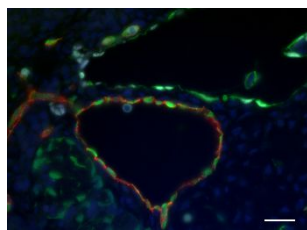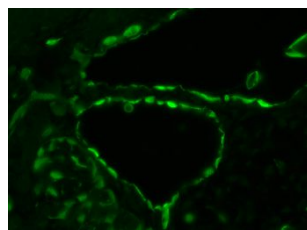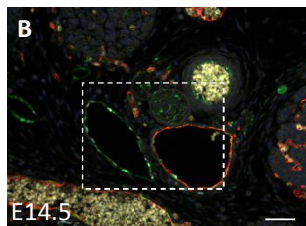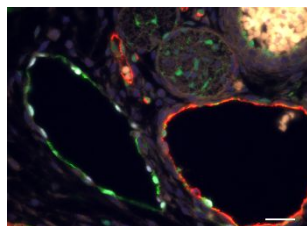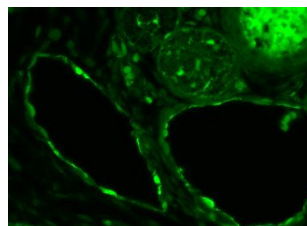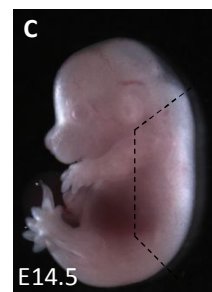

Supplement: Additional file 6: Figure S4. — BRE::gfp transcriptional activity is similar in blood and lymphatic vessels. Transverse sections through the neck of E12.5 (A) and E14.5 (B) BRE::gfp embryos immunostained for GFP, Endomucin, PROX1 and DAPI. Boxed areas are enlarged in the right panels with the single GFP staining next to it. (C) Schematic overview shows the dorsal skin area taken from an E14.5 embryo. Scale bars: 50 μm (A-B, left panels); 25 μm (A–B, right panels). (PDF 136 kb) [file 12861_2016_133_MOESM6_ESM.pdf]

$\alpha$ GFP  $\alpha$ CollagenIV  $\alpha$ PROX1

Skin biopsy

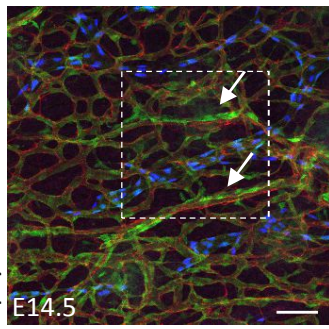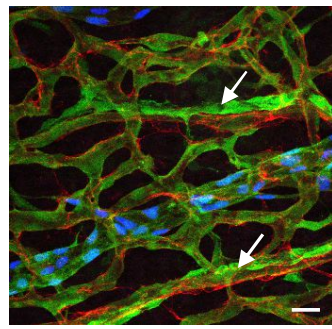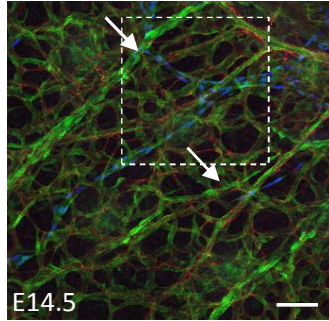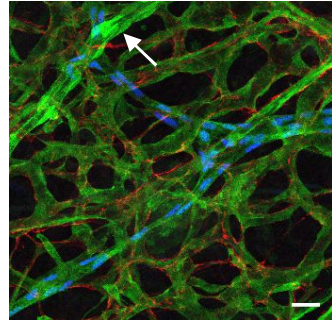

Supplement: Additional file 7: Figure S5. — BRE::gfp transcriptional activity is not restricted to vessels. E14.5 skin biopsies with immunodetection of GFP, PROX1 and the extracellular matrix marker Collagen type IV. Boxed areas are enlarged in the right panels. Arrows show non-vessel structures. Scale bars: 75 μm (left panels); 25 μm (right panels). (PDF 184 kb) [file 12861_2016_133_MOESM7_ESM.pdf]

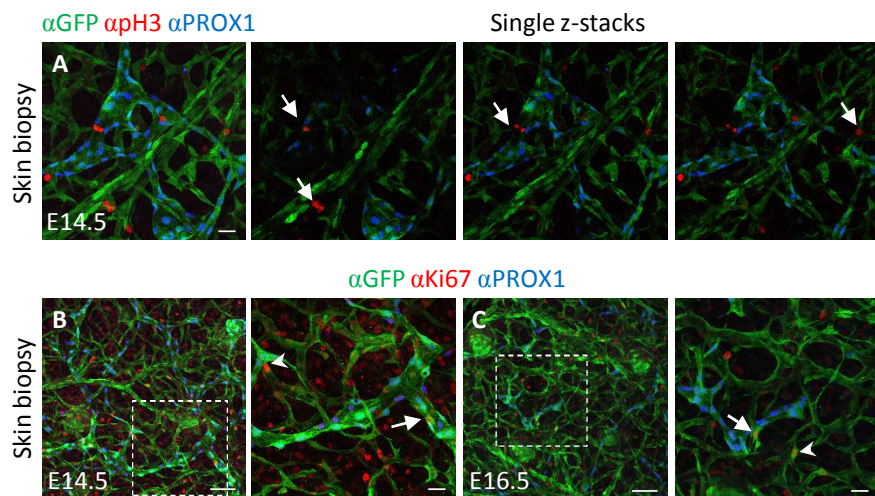

Supplement: Additional file 8: Figure S6. — BRE::gfp transcriptional activity does not co-localise with Ki67 in ECs. (A) Skin biopsy of an E14.5 BRE::gfp mouse with immunodetection of GFP, pH3 and PROX1. The merged stack in the left panel is split up in some single z-stacks to show pH3-positive cells beneath or above vessel structures (arrows). E14.5 (B) and E16.5 (C) skin biopsies with immunodetection of a different proliferation marker (Ki67), GFP and PROX1. Boxed areas are enlarged in the right panels. Ki67-positive LECs (arrows) and blood ECs (arrowheads) can be observed. Scale bars: 75 μm (B–C, left panels); 25 μm (A; B–C, right panels). (PDF 184 kb) [file 12861_2016_133_MOESM8_ESM.pdf]
