# Supplementary material for: BMP-SMAD signalling output is highly regionalized in cardiovascular and lymphatic endothelial networks
Source: BMC Dev Biol. 2016 Oct 10;16:34. doi: 10.1186/s12861-016-0133-x (PMC5057272; doi:10.1186/s12861-016-0133-x)
Supplement: Additional file 4: Table S2. — GFP localisation patterns in embryonic hearts with emphasis on BRE::gfp activity in endocardial cells. The number of hearts analysed is indicated in the top row. Results are consistent for all hearts analysed, unless specifically indicated. (C) Continuous (100 %); (U) Ubiquitous (>70 %); (M) Mosaic (10–70 %); (O) Occasional cell (<10 %); (P) Patches; (ND) undetectable; N/A not applicable (PDF 215 kb) [file 12861_2016_133_MOESM4_ESM.pdf]

Endocardial cells	E9.5 N=3	E10.5 N=6	E11.5 N=3-6	E12.5 N=3	E14.5 N=3	E16.5 N=3
**Aortic Sac**	M (2/3)	M	M	N/A	N/A	N/A
**Truncal region**	M	M	M	N/A	N/A	N/A
**Common atrium**	U (2/3)	N/A	N/A	N/A	N/A	N/A
**Dorsal Atrium**	N/A	O	O	Increasing activity	M	M
**Ventral Atrium**	N/A	O	O	O	N/A	N/A
**Ventricles**	M	M	M	M	M	M
**IVS**	N/A	N/A	O	O	M	M
**Sinus venosus horns**	U (2/3)	U	U	U	N/A	N/A
**Inflow valves**	N/A	N/A	U (5/6)	U (2/2)	M	M
**Atrial septum**	N/A	U	U	M	M	M
**Superior / Inferior cushions**	M	M	M	M	N/A	N/A
**Lateral cushions**	N/A	N/A	O	O	N/A	N/A
**AV valves**	N/A	N/A	N/A	M	P (atrial side)	P (atrial side)
**Proximal OFT**	N/A	M	M	N/A	N/A	N/A
**Distal OFT**	N/A	M	M	N/A	N/A	N/A
**Pulmonary valve**	N/A	N/A	M	M	M	M
**Aortic valve**	N/A	N/A	M	M	M	M

Non-endocardial cells	E9.5 N=3	E10.5 N=6	E11.5 N=3-6	E12.5 N=3	E14.5 N=3	E16.5 N=3
**AV myocardium**	M	M	M	M	M	N/A
**Bulbo-Ventricular myocardium**	U	ND	ND	N/A	N/A	N/A
**Cushion mesenchyme**	N/A	O	O	ND	ND	ND
**OFT mesenchyme**	N/A	ND	ND	ND	N/A	N/A
**Epicardium**	N/A	M	M	M	M	M
